# Posterior Reversible Encephalopathy Syndrome, not so Uncommon in Pediatric Patients with Renal Involvement: A Case Series

**DOI:** 10.2478/jccm-2024-0004

**Published:** 2024-01-30

**Authors:** Ana-Maria Roxana Koller, Alexandra Man, Carmen Muntean

**Affiliations:** Department of Pediatrics, County Emergency Clinical Hospital Targu Mures, Romania; George Emil Palade University of Medicine, Pharmacy, Science, and Technology of Targu Mures, Romania

**Keywords:** posterior reversible encephalopathy syndrome, renal involvement, clinico-radiological findings, children

## Abstract

**Introduction:**

Posterior reversible encephalopathy syndrome (PRES) primarily shows neurological symptoms and is more frequent in males, often occurring in oncological patients. It can also be associated with renal conditions like post-streptococcal glomerulonephritis, a common cause of pediatric hypertension. Management involves blood pressure and seizure treatment. In some cases, it may lead to irreversible and severe complications. Early treatment is essential for prevention.

**Presentation of case series:**

In the past six months, we have documented the cases of two patients, aged 15 and 10, both of whom presented with PRES and renal disease. These patients were admitted because of general malaise, headaches, nausea, vomiting, visual disturbances, and elevated blood pressure. Subsequently, both patients experienced epileptic episodes. Only the first patient required transfer to the Pediatric Intensive Care Unit (PICU). Cerebral magnetic resonance imaging (MRI) scans revealed distinct PRES lesions in both cases. Following comprehensive investigations, both cases were diagnosed with PRES in the context of acute post-streptococcal glomerulonephritis.

**Conclusions:**

The patients showed improvement following the administration of antihypertensive and anticonvulsant medications, along with treatment for the underlying renal condition.

## Introduction

Posterior Reversible Encephalopathy Syndrome (PRES) primarily manifests as neurological issues including seizures, altered consciousness, intense headaches, nausea, vomiting, and visual disturbances. It is more prevalent among males [1,2,3,4]. While it is frequently observed in oncological patients, specialized literature indicates its association with conditions such as acute glomerulonephritis, end-stage chronic kidney disease, renal artery stenosis, hemolytic-uremic syndrome, or lupus nephritis [2,5,6].

Hence, the most frequently encountered factors that increase the risk of PRES include arterial hypertension, renal disorders, and the use of calcineurin inhibitors, like Cyclosporine A, Methotrexate, Tacrolimus, Metronidazole and Vigabatrin. Discontinuing immunosuppressive treatment could potentially reverse the neurological symptoms in children with PRES [7,8,9]. Contributing to the development of PRES are electrolyte imbalances like hypomagnesemia and hypercalcemia, elevated cholesterol levels, high doses of Methylprednisolone, the use of non-steroidal anti-inflammatory drugs (NSAIDs), blood transfusions, erythropoietin therapy, and the presence of human immunodeficiency virus (HIV) infection [10].

Two theories explain the occurrence of cerebral vasogenic edema from a pathophysiological perspective: hyperperfusion due to impaired autoregulation of cerebral blood flow and hypoperfusion resulting from vasoconstriction of cerebral arteries [10]. As the brain matures, the autoregulation mechanism of cerebral perfusion improves. However, during episodes of increased blood pressure in children, this mechanism becomes more susceptible to vasoconstriction. Additionally, PRES can occur as a result of systemic inflammation, leading to endothelial dysfunction in cerebral vessels [11,12].

Typically, diagnosis relies on imaging techniques. Cerebral edema is often identified through hyperintense lesions seen in magnetic resonance imaging (MRI) scans, specifically in transverse relaxation time (T2) and fluid-attenuated inversion recovery (FLAIR) sequences. These lesions indicate swelling in the cerebral white matter, commonly affecting the frontal, parietal, and occipital lobes symmetrically. However, in some cases, atypical lesions can occur in areas like the brainstem, cerebellum, basal ganglia, internal capsule, and corpus callosum [3,4,13,14].

The primary cause of hypertension among pediatric patients is kidney parenchymal disorders, like acute glomerulonephritis. This condition typically manifests gradually, with limited clinical symptoms, and often less prominent edema. According to most literature findings, less than half of children affected by this condition exhibit visible macroscopic hematuria [15]. Treatment includes regulating blood pressure, controlling seizures and addressing the root cause of the disease, discontinuing triggering medications, and rectifying electrolyte and acid-base imbalances [1,14]. While the prognosis is usually positive, patients need ongoing, long-term monitoring and care [7,8,9,10,11,12,13]. Moreover, it’s essential to note that PRES can lead to irreversible consequences, and in some cases, patients may experience severe, life-threatening complications such as transforaminal cerebellar herniation, which can result in focal neurological deficits. To prevent such outcomes, it is crucial to promptly initiate treatment aimed at addressing the underlying causes that can give rise to this syndrome [16].

This case series aims to outline the clinical symptoms and alterations in paraclinical measures in two pediatric patients with both renal impairment and PRES, emphasizing potential complications in the absence of targeted treatment. Establishing a prevention plan is crucial, considering its impact on the patient’s quality of life. Equally significant is the follow-up care and complete recovery of PRES patients, underscoring the importance of comprehensive, long-term management.

## Presentation of case series

### Case 1

A male adolescent, 15 years old, who is overweight and of short stature, with a recent history of upper respiratory tract infection, was admitted with fever (peaking at 38°C), along with symptoms of nausea, vomiting, headache, oliguria and elevated blood pressure (150/100 mmHg) that had persisted for three days. His condition deteriorated rapidly, with blood pressure surging to as high as 197/111 mmHg, and his oxygen saturation dropped to 60% in ambient air. He also experienced two seizures while afebrile, initially presenting as focal on the left side and later becoming generalized, prompting the administration of intravenous Diazepam.

Laboratory assessments indicated the presence of anemia, and leukocytosis with an increase in neutrophils, hyperglycemia, and low albumin levels (3.3 g/L). A cranial computed tomography (CT) scan revealed a subacute/chronic hypodense lesion on the right frontal region without contrast enhancement. Additionally, there was a slight asymmetry in the ventricular system at the midline and small hypodense areas near the occipital horn of the right lateral ventricle.

After consultation with a neurosurgeon, it was advised to undergo a cerebral MRI. Additionally, a cardiology specialist recommended maintaining systolic blood pressure within the range of 140–150 mmHg, while investigating the underlying cause of hypertension and reevaluating the need for further intervention.

Following this, the patient was admitted to the Pediatric Intensive Care Unit (PICU) and underwent intubation and mechanical ventilation. Both urine and blood cultures yielded negative results. Furthermore, the inflammatory marker values were found to be within the expected normal ranges. Urine analysis revealed the presence of microscopic hematuria (Table 1).

**Table 1.** Urine analysis and immunology tests performed on the third day following admission of the patients to the pediatric nephrology department

**Analysis**	**Case 1**	**Case 2**
Urinalysis		
Erythrocytes	300/μL	300/μL

Urine sediment		
Erythrocytes	11–20/field	21–50/field
	80% dysmorphic red blood cells, 20% isomorphic red blood cells /field	95% dysmorphic red blood cells, 5% isomorphic red blood cells /field
	Hematic casts	
Leukocytes	0–5/field	0–5/field
Proteins	negative	1 g/L
C3	0,33 g/L	0,19 g/L
C4	0,104 g/L	0.123 g/L
C3 after 6 weeks	1,48 g/L	1,17 g/L

C3 – Complement 3; C4 – Complement 4.

Given the presence of leukocytosis and neutrophilia, a 10-day treatment regimen was initiated. This regimen involved the intravenous administration of Ceftriaxone, as well as the utilization of Furosemide to promote diuresis and Urapidil for blood pressure reduction. Additionally, adjustments were made to address electrolyte and acid-base levels, and a central venous catheter was inserted.

Following consultation with an infectious disease specialist and considering a potential diagnosis of acute encephalitis, the treatment strategy was adjusted. This adjustment included raising the intravenous dose of Ceftriaxone to 2 grams twice daily and introducing Dexamethasone to address the inflammatory process. Osmofundin was added to address depletion, and Enalapril was included to enhance the effectiveness of the antihypertensive therapy.

After the neurological consultation, it was advised to start antiepileptic therapy with Phenobarbital, and an electroencephalogram (EEG) was performed, that revealed no focal anomalies.

**Fig. 1.**
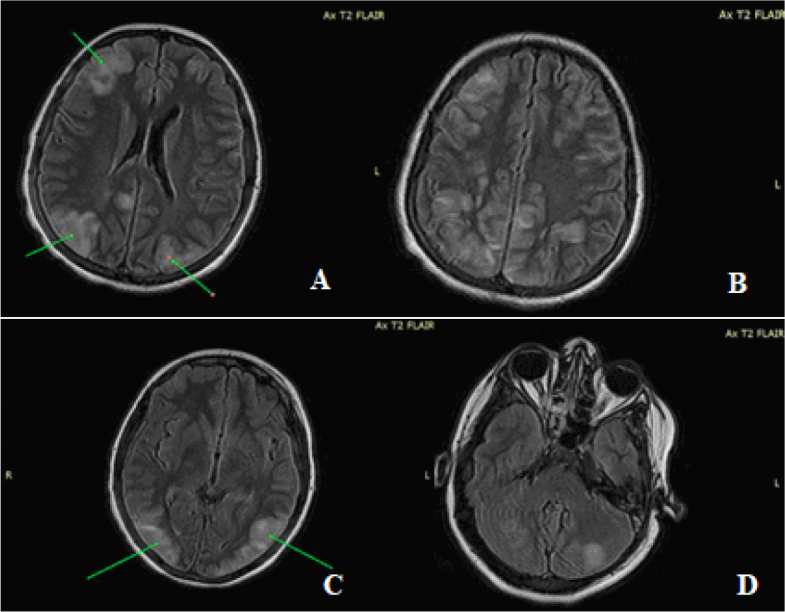
Multiple infra and supra centimeter lesions in hypersignal T2 and FLAIR diffusely arranged bihemispheric cortico-subcortical in (A, B) frontal and parietal lobes, (C) occipital lobe and (D) bihemispheric cerebellar

A cranial MRI showed several lesions, both infra and supra-centimeter in size, appearing hyperintense on T2 and FLAIR, and isointense on longitudinal relaxation time (T1), with unrestricted diffusion. These lesions were symmetrically distributed in the bilateral cortical-subcortical hemispheres and cerebellar regions, indicating inflammatory activity (Figure 1.). Given the patient’s clinical state and the MRI findings, the lumbar puncture was delayed.

Throughout the treatment, the patient’s blood pressure remained at approximately 150/88 mmHg. Subsequently, a 24-hour urinary metanephrine test (Normetanephrine: 73 μg/24 h; 0.4 μmol/24 h, Urine metanephrines: 85.8 μ/24 h; 0.44 μmol/24 h) yielded normal results, ruling out the possibility of pheochromocytoma.

Due to the patient’s unchanging condition, the treatment plan was modified to include Immunoglobulin, a 10-day course of Acyclovir, prophylactic Clexane, and Spironolactone. Additionally, Clindamycin (administered for 9 days) was introduced to address a broader spectrum of potential microbial causes, along with antifungal medication.

After two days, the patient was safely taken off the ventilator, displaying stable respiratory and hemodynamic conditions, but the patient’s blood pressure remained consistently elevated, so he was referred to the nephrology department. Here, the suspicion of acute glomerulonephritis and PRES was raised, considering the patient’s medical history, reduced urine output, presence of blood in the urine, and MRI findings.

In light of the elevated blood pressure readings, an ophthalmological assessment was considered, but no abnormal findings were observed. Following this, a more robust approach to blood pressure management, involving Amlodipine and Lisinopril, was implemented, resulting in the stabilization of blood pressure within the normal range for the child’s height and gender (ranging from 110/50 to 123/77 mmHg). Additionally, aside from elevated transaminase levels, an abdominal ultrasound revealed only mild hepatomegaly.

Tests for interleukin-6 (IL-6), erythrocyte sedimentation rate (ESR), and the immunological panel showed results within the normal range.

According to the medical history of the patient, an Antistreptolysin O (ASO) test was done, which returned elevated values (> 800 IU/ml). A dynamic urine analysis consistently indicated microscopic hematuria, with the urinary sediment revealing a significant proportion of dysmorphic red blood cells and erythrocyte casts. Additionally, complement 3 (C3) and complement 4 (C4) levels were assessed, and C3 displayed low values (Table 1).

Therefore, the diagnosis of acute post-streptococcal glomerulonephritis and PRES resulting from renal hypertension with neurological implications was confirmed through the correlation of anamnesis, clinical and paraclinical information. Given the patient’s overall well-being and stable blood pressure, the boy was discharged with instructions to follow the low-sodium diet, regularly monitor blood pressure, continue anti-hypertensive medication (Amlodipine), conduct C3 measurements six weeks after the initial assessment, undergo a cerebral MRI after six months, continue with antiepileptic therapy, and undergo subsequent neurological evaluations.

### Case 2

A 10-year-old girl was admitted to a territorial hospital with a fever, rhinorrhea, and a severe headache. However, after 48 hours, her condition worsened. She developed central dyspnea, the Acid-base balance test revealed hypercapnia, and her blood pressure increased to 160/100 mmHg. This was followed by a subsequent epileptic episode, for which she received corticosteroids, anticonvulsants (Diazepam), diuretics, and depletive treatment.

The patient exhibited a spastic cough, and the clinical examination revealed the presence of sibilant rales. Consequently, a chest X-ray was conducted, revealing localized pneumonia. As a result, treatment with Dexamethasone and Ventolin was initiated.

In order to exclude the possibility of a tumor or acute meningoencephalitis, a cranial CT scan was performed. However, the scan revealed pansinusitis and left otomastoiditis, leading to the initiation of antibiotic treatment with Meropenem. Furthermore, lumbar punctures were performed and did not indicate any pathological changes.

In a span of 24 hours, the patient noted visual disturbances. Subsequent urine analysis showed both macroscopic hematuria and proteinuria, raising suspicions of a potential nephritic syndrome. Considering the patient’s elevated blood pressure, renal issues, and a prior episode of convulsions, there is a possibility of a diagnosis of PRES being taken into account.

As the patient’s condition deteriorated, she was relocated to the pediatric nephrology department. The patient exhibited altered mental status, drowsiness, pallor, slight eyelid edema, bradycardia (45 beats per minute), and had a blood pressure reading of 135/85 mmHg. Additionally, the patient experienced symptoms like headaches and visual disturbances, leading to the administration of Amlodipine and Clonidine.

**Fig. 2.**
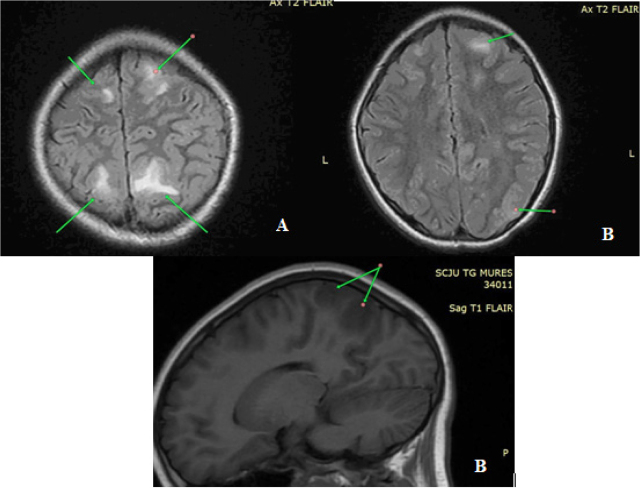
An inflammatory cerebral lesion showing hypersignal on T2 and FLAIR sequences, located at the level of the bilateral frontal lobe and post-central gyrus (A), respectively on the left parietal side (B)

A neurological evaluation was conducted, and EEG testing was carried out, with Diazepam prescribed when needed. During the ophthalmological examination, slightly pale papillae and thinner retinal vessels were observed. A cranial MRI confirmed the diagnosis of PRES and left mastoiditis by showing inflammatory brain lesions (Figure 2).

The patient received a cardiology exam and cardiac ultrasound, which identified a small amount of fluid in the pericardium with no significant hemodynamic impact. It was advised to monitor vital signs, continue antihypertensive and diuretic treatment to manage blood pressure and reevaluate as necessary.

While attempting to gradually reduce the Clonidine dosage, it was observed that the patient’s blood pressure readings increased, necessitating a return to the initial dose. The patient’s condition improved gradually, characterized by a reduction in headache and the resolution of visual disturbances. Nevertheless, elevated blood pressure levels persisted.

Dynamic urine analyses indicated microscopic hematuria (300/μL erythrocytes) with proteinuria (1,8 g/24 h), despite laboratory results indicating hypoalbuminemia (3.08 g/dL). The ASO level was elevated (1200 IU/ml), and C3 and C4 were assessed, revealing low C3 values (Table 1.).

Upon release, the diagnosis was established as acute post-streptococcal glomerulonephritis associated with PRES. Upon discharge, the patient’s improvement was quite notable, although the blood pressure remained at 120/80 mmHg. It is recommended to maintain regular blood pressure monitoring and make any essential adjustments to the antihypertensive treatment (Amlodipine, Captopril, Clonidine). Furthermore, it is suggested that an EEG be repeated in one month, and a follow-up cranial MRI be scheduled over six months.

Both patients had a good evolution, with C3 values returning to normal after six weeks and blood pressure levels remaining within normal ranges. After a 6-month MRI examination revealed no more alterations, the decision was made to discontinue anticonvulsant treatment due to the lack of clinical symptoms and controlled blood pressure.

## Discussions

Assessing patients with kidney diseases such as acute post-streptococcal glomerulonephritis, nephrotic syndrome, acute kidney injury, and end-stage renal disease reveals a significantly increased risk of developing hypertension and, consequently, PRES [1,13]. In cases of renal involvement, hypoalbuminemia and substantial proteinuria play pivotal roles in the pathogenesis of PRES [2,18]. In our cases, hypoalbuminemia was evident, with transient proteinuria (proteinuria disappeared after two weeks), observed only in the second case.

Contrary to the findings by Siebert et al., who linked increased C-reactive protein (CRP) values and sepsis to higher mortality in the first episode of PRES [1], our case series displayed normal CRP and ESR values.

Tai Heng Chen et al. demonstrated that arterial hypertension is present in over 80% of pediatric cases, serving as an important warning sign for the onset of PRES [10,11]. Similarly, Donmez and Onder et al. reported that hypertension is a trigger for recurrent PRES in patients with advanced chronic kidney disease [1]. In our clinical cases, increased blood pressure was the initial manifestation leading to PRES in both patients.

Seizures, encephalopathy, and headaches are among the most common symptoms in patients with PRES at the time of presentation. Some studies have highlighted encephalopathy and seizures as characteristics of PRES [1,18]. In our patients, symptoms before admission included altered general condition, nausea, vomiting, lipothymia, seizures (status epilepticus), and visual disturbances. Status epilepticus has been associated with the need for transfer to the PICU and can be a severe complication leading to fatality [1,5]. The development of secondary epilepsy in PRES has been reported in up to 15% of children of all ages [1]. Some studies suggest that status epilepticus is more common in young female pediatric patients due to fewer inter-neuronal connections and greater diffusivity in the parieto-occipital area compared to boys. Additionally, children under 6 years old appear to have a higher frequency of status epilepticus than older children [5]. Cordelli et al. also showed that cerebral hemorrhage, cerebellar herniation, and refractory status epilepticus can occur at the onset of PRES [17]. Patients who present with status epilepticus tend to have longer PICU hospitalizations and a higher risk of developing secondary epilepsy [5]. In our research, both patients experienced seizures, but only the first patient required a 5-day stay in the PICU.

The early diagnosis of this condition relies on recognizing and correlating clinical and radiological findings. The typical MRI appearance of PRES includes symmetric hyperintense lesions, predominantly in the parietal and occipital lobes. CT scans can help detect intracranial hemorrhages [18]. Clinically and radiologically, our patients exhibited typical, symmetric hyperintense lesions located in the frontal, parietal, and occipital lobes.

## Conclusions

Individuals with kidney impairment face an elevated risk of PRES development owing to heightened arterial hypertension. Timely recognition of symptoms and accurate interpretation of cerebral MRI scans are crucial for prompt treatment. The prognosis hinges on initial symptoms, the type of MRI lesions (typical or atypical), and subsequent neurological issues. Regular clinical and radiological follow-ups are vital to prevent recurrence and severe complications. Our series of cases emphasizes the necessity of regarding PRES as a potential diagnosis in instances of hypertension, seizures, and renal issues. Adequate management and sustained, long-term monitoring are pivotal. Therefore, this case series stands out due to the age at which the syndrome occurs and its connection with acute post-streptococcal glomerulonephritis in two patients who do not have significant personal medical histories.

## Ethics

This case series does not require committee approval and does not include any identifiers of the patient to protect confidentiality. Written informed consent was obtained from the patient’s parents with their agreement to the publication of this case series.

## Abbreviations

ASO: antistreptolysin O
C3: complement component 3
C4: complement component 4
CRP: C-reactive protein
CT: computed tomography
EEG: electroencephalogram
ESR: erythrocyte sedimentation rate
FLAIR: fluid-attenuated inversion recovery
HIV: human immunodeficiency virus
IL-6: interleukin 6
MRI: magnetic resonance imaging
NSAIDs: non-steroidal anti-inflammatory drugs
PICU: Pediatric Intensive Care Unit
PRES: Posterior Reversible Encephalopathy Syndrome
T1: longitudinal relaxation time
T2: transverse relaxation time
